# Neurofibromatosis: Molecular Pathogenesis and Natural Compounds as Potential Treatments

**DOI:** 10.3389/fonc.2021.698192

**Published:** 2021-09-17

**Authors:** Anusha Amaravathi, Janet L. Oblinger, D. Bradley Welling, A. Douglas Kinghorn, Long-Sheng Chang

**Affiliations:** ^1^Center for Childhood Cancer and Blood Diseases, Abigail Wexner Research Institute at Nationwide Children’s Hospital, Columbus, OH, United States; ^2^Department of Medicine, University of Rochester School of Medicine and Dentistry, Rochester, NY, United States; ^3^Department of Otolaryngology Head & Neck Surgery, Harvard Medical School, Massachusetts Eye and Ear, and Massachusetts General Hospital, Boston, MA, United States; ^4^Division of Medicinal Chemistry and Pharmacognosy, The Ohio State University College of Pharmacy, Columbus, OH, United States; ^5^Department of Pediatrics, The Ohio State University College of Medicine, Columbus, OH, United States; ^6^Department of Otolaryngology-Head & Neck Surgery, The Ohio State University College of Medicine, Columbus, OH, United States

**Keywords:** neurofibromatosis (NF), signaling pathway, targeted therapy, natural compounds, eIF4A inhibitors, rocaglamide, didesmethylrocaglamide, protein translation

## Abstract

The neurofibromatosis syndromes, including NF1, NF2, and schwannomatosis, are tumor suppressor syndromes characterized by multiple nervous system tumors, particularly Schwann cell neoplasms. NF-related tumors are mainly treated by surgery, and some of them have been treated by but are refractory to conventional chemotherapy. Recent advances in molecular genetics and genomics alongside the development of multiple animal models have provided a better understanding of NF tumor biology and facilitated target identification and therapeutic evaluation. Many targeted therapies have been evaluated in preclinical models and patients with limited success. One major advance is the FDA approval of the MEK inhibitor selumetinib for the treatment of NF1-associated plexiform neurofibroma. Due to their anti-neoplastic, antioxidant, and anti-inflammatory properties, selected natural compounds could be useful as a primary therapy or as an adjuvant therapy prior to or following surgery and/or radiation for patients with tumor predisposition syndromes, as patients often take them as dietary supplements and for health enhancement purposes. Here we review the natural compounds that have been evaluated in NF models. Some have demonstrated potent anti-tumor effects and may become viable treatments in the future.

## Neurofibromatosis (NF) Comprises Three Distinct Genetic Disorders That Cause Tumors to Grow Along Nerves

As a group of slowly progressive autosomal-dominant syndromes, NF is classified into neurofibromatosis type 1 (NF1), neurofibromatosis type 2 (NF2), and schwannomatosis (1). These syndromes typically present with neural tumors, which manifest in different locations depending upon their genetic etiology. Although the tumors frequently remain benign, they incur severe patient morbidity and occasionally exhibit malignant progression.

NF1, previously known as von Recklinghausen disease, affects ~1 in 3,000 individuals (2) and is caused by mutations of the *NF1* gene on chromosome 17q11.2 (3–5). Although *NF1* mutations can be inherited, the *de novo* mutation rate is relatively high (~42%). With 100% penetrance, NF1 patients are symptomatic, but with variable expression. First presenting with hyperpigmentation-like café-au-lait macules and freckling in afflicted patients, the hallmark of NF1 is the development of neurofibromas, benign tumors composed of an admixture of dysplastic Schwann cells and stromal cells, including fibroblasts, mast cells, and perineural cells embedded in a collagenous extracellular matrix (ECM). Nearly all NF1 patients develop cutaneous neurofibromas (CNFs, or dermal neurofibroma), which arise along superficial nerves and are confined to the cutaneous tissues (6). CNFs can cause itching and pain. About half of NF1 patients also develop more serious plexiform neurofibromas (PNFs) (7). These tumors occur deeper within the body and are more extensive, surrounding multiple nerve roots and causing pain and disfigurement. PNFs can undergo malignant progression, and 8-13% of PNFs in NF1 patients develop into highly-aggressive malignant peripheral nerve sheath tumors (MPNSTs) (8).

A fraction (15-20%) of NF1 patients develop optic pathway gliomas (OPGs, astrocytomas of the optic tract) (9). Patients with OPGs may present with visual disturbances and progressive vision loss. NF1 patients are also predisposed to other types of tumors, including gliomas, gastrointestinal stromal tumors, juvenile myelomonocytic leukemia, and glomus tumors (10). Although most NF1-related gliomas are classified as benign pilocytic astrocytomas, adult NF1 patients are at ~50-fold increased risk of developing malignant glioblastomas.

For current NF1 treatment, surgical excision of CNFs can be performed when symptomatic (6). Surgical removal of PNFs is more challenging because they are more diffuse and involve multiple nerve roots. A recent exciting development is the FDA approval of the MEK inhibitor selumetinib (Koselugo^™^) for the treatment of children with symptomatic, inoperable PNF based on the trial (ClinicalTrials.gov Identifier: NCT01362803) which demonstrated a 72% response rate in tumor volume shrinkage by ≥20% in NF1 children with PNFs (11). However, this is not curative as no subjects had complete tumor disappearance and tumors regrew particularly after dose reduction or cessation due to toxic effects, including diarrhea, weight gain, paronychia, skin ulceration, and elevated creatinine level. Identification of other targeted agents that synergize with selumetinib in eliminating PNFs should be sought. Several other MEK inhibitors, such as trametinib (Mekinist^®^; ClinicalTrials.gov Identifiers: NCT02124772 and NCT03741101) and cobimetinib (NCT02639546), are also being evaluated in children and adults with PNFs. Mirdametinib was recently reported to shrink the sizes of PNFs in adults and reduce tumor-associated pain (NCT02096471) (12), and a larger trial of this drug in children and adults with NF1 is currently recruiting (NCT03962543). Additionally, a topical gel formulation of the MEK inhibitor NFX-179 is being tested in NF1 patients with CNFs (NCT04435665).

Children with OPGs are routinely monitored for clinical progression by neuroimaging and visual acuity tests (13). Slow-growing gliomas causing minimal symptoms are usually not treated, but if the disease progresses, chemotherapy and occasionally, surgery is used to stabilize and reduce tumor burden. Radiation is contraindicated in NF1 patients with benign tumors due to the heightened risk of secondary malignancies. In a phase 2 clinical trial of selumetinib for pediatric low-grade glioma, sustained responses were seen in ~40% of NF1 patients (14), leading to a phase 3 trial comparing selumetinib to chemotherapy (ClinicalTrials.gov Identifier: NCT03871257). Due to highly aggressive behavior, MPNSTs are excised with wide margins (8). Radiation may be used following resection of large (≥5cm) tumors, and chemotherapy can be applied in a primary or adjuvant setting to treat unresectable or metastatic tumors. Despite these efforts, the local recurrence rate remains high (~32-65%), suggesting an urgent need for additional effective treatments (8, 15).

NF2 has an incidence of ~1 in 30,000 and has nearly complete penetrance (16). It is caused by mutations in the *NF2* gene on chromosome 22q12.2 (17, 18). The hallmark of NF2 is bilateral vestibular schwannomas (*VS*). As the most common benign tumors of the cerebellopontine angle, 95% of *VS* are unilateral and occur sporadically. Occasionally, unilateral tumors are found in NF2 patients, especially when they are mosaic for *NF2* loss. Like NF2-related *VS*, sporadic unilateral *VS* harbor *NF2* mutations (19). Compared to sporadic *VS*, NF2-associated tumors display more aggressive behavior, with a propensity towards multifocal, rapid growth. Due to their intracranial location and proximity to cranial nerves, *VS* present with serious comorbidities, including hearing loss, tinnitus, balance dysfunction, facial weakness, and brainstem compression. NF2 is also associated with an increased incidence of cutaneous schwannomas.

Patients with NF2 also develop meningiomas and less commonly, spinal schwannomas, ependymomas, and astrocytomas (1, 16). Meningiomas are the most common brain tumors, and ~80% are benign (WHO grade I), whereas the remaining are atypical (grade II) and anaplastic (grade III) (20). In addition to NF2-related meningiomas, *NF2* mutations are found in ~50% of sporadic meningiomas. Up to 60% of NF2 patients develop meningiomas, mostly benign and often multiple, which are associated with disease severity and increased mortality (21). These tumors cause significant morbidity, including cranial nerve palsy, seizures, and brainstem compression, which may lead to paralysis, aspiration pneumonia, and death.

The current standard-of-care for NF2-related tumors is surgery (1, 16, 19). Radiosurgery, such as stereotactic γ-knife radiation, may be used, especially for unresectable tumors. However, it must be weighed against the risk of causing malignant transformation of benign tumors and inducing second-site malignancies. Also, surgery may not be recommended if the tumor is located in critical structures or if there is high tumor burden (21). Currently, an FDA-approved medical therapy is not available for NF2 patients, although a few targeted therapies have been evaluated (1). Bevacizumab (Avastin^®^), a monoclonal antibody that neutralizes vascular endothelial growth factor (VEGF), is administered off-label to patients with NF2 and progressive *VS*, with ~36-41% of the patients experiencing durable hearing improvement and reduced tumor volume (22, 23). However, intravenous administration of bevacizumab is needed, and its adverse effect profile, including hypertension and proteinuria, may preclude long-term use for some patients. Also, meningiomas are unresponsive to bevacizumab (24). Another targeted drug, the kinase inhibitor brigatinib, is currently in a phase 2 clinical trial for NF2-related tumors (ClinicalTrials.gov Identifier: NCT04374305).

Schwannomatosis manifests as multiple schwannomas on nerves throughout the body but without involvement of vestibular nerves (1, 25). The true incidence of schwannomatosis is unknown but thought to be similar to NF2. Schwannomatosis usually occurs sporadically, although familial or mosaic cases occasionally occur. The most common familial mutations occur in either the *SNF5*/*SMARCB1*/*INI1* (Switch/Sucrose Non-Fermentable chromatin remodeling complex subunit-5/SWI/SNF-related, Matrix-associated, Actin-dependent Regulator of Chromatin, subfamily-B, member-1/INtegrase Interactor 1) gene on chromosome 22q11.23 or the *LZTR1* (Leucine Zipper-like Transcriptional Regulator 1) gene on chromosome 22q11.21; both are located near the *NF2* locus (26, 27). Interestingly, schwannomatosis-related schwannomas also exhibit *NF2* inactivation. It is currently thought that familial schwannomatosis is inherited through a “three-event, four-hit” process where inactivating mutations occur in the *NF2* gene located on the same chromosome as the germline-mutated *LZTR1* or *SMARCB1* allele. Loss of the remaining normal copy of chromosome 22q results in biallelic loss-of-heterozygosity (1, 16). However, some patients with schwannomatosis do not have *LZTR1* or *SNF5/SMARCB1/INI1* mutations, suggesting the presence of another tumor suppressor gene on chromosome 22q in schwannomatosis development.

Patients with schwannomatosis are often diagnosed at age over 30 and frequently present with chronic debilitating pain. As an FDA-approved drug is also not available, surgery is considered for symptomatic patients. As pain is the major symptom experienced by patients with schwannomatosis, the anti-nerve growth factor (NGF) neutralizing monoclonal antibody tanezumab is being tested in a phase 2 trial for pain alleviation (ClinicalTrials.gov Identifier: NCT04163419). Schwannomatosis is also associated with an increased risk of meningioma (1, 16). Unlike NF2, schwannomatosis patients with germline loss of *SMARCB1* have a higher risk of developing MPNSTs.

## Signaling Pathways Affected in NF-Associated Tumors

**Figure 1:**
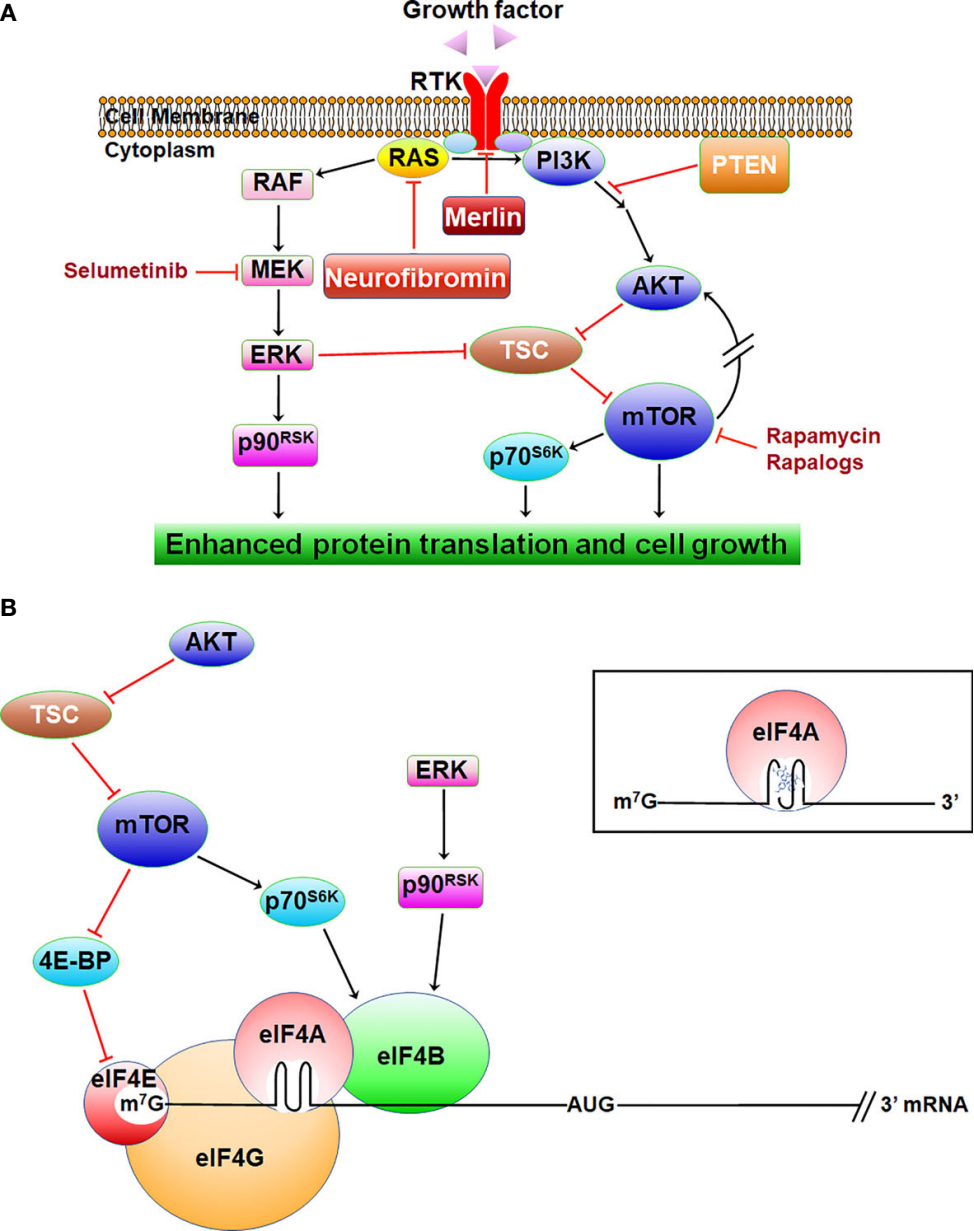
The signaling pathways inhibited by neurofibromin and merlin. **(A)** NF-related tumors exhibit activated Ras/RAF/MEK/ERK and PI3K/AKT/mTOR, leading to enhanced protein translation and growth. **(B)** Activated ERK and AKT/mTOR enhance translation *via* eIF4B, which stimulates eIF4A activity. AKT/mTOR also promotes translation *via* eIF4E. Inset: rocaglamide locks eIF4A onto the structured 5’-UTR of mRNA.

The *NF1* gene encodes neurofibromin, a Ras-GAP (GTPase-activating protein) which stimulates the intrinsic GTPase activity of Ras, converting it from the active GTP-bound to inactive GDP-bound state (9, 28). Therefore, *NF1*-deficient cells consistently exhibit excessive levels of Ras-GTP and activation of its downstream signaling, including the Raf/MEK/ERK mitogen-activated protein kinase (MAPK) and phosphoinositide-3-kinase (PI3K)/AKT/mammalian target of rapamycin (mTOR) pathways (**Figure 1**). Activated ERKs phosphorylate several effectors important for cell proliferation, including components of the protein translation apparatus, cell-cycle proteins, transcription factors, other kinases, and phosphatases. Activated AKT signals multiple downstream targets, promoting cell growth, survival, and motility (29). Through phosphorylation of mTOR, AKT also promotes protein translation. The mTOR protein functions as a component in two multi-protein complexes, mTORC1 and mTORC2, with overlapping protein compositions but distinct cellular functions (30). By integrating various extracellular and intracellular signals, including growth factor receptor signaling and the levels of ATP, oxygen, and nutrients, mTORC1 regulates ribosome biogenesis and protein translation. The mTORC2 complex is insensitive to nutrient levels but is responsive to growth factor-mediated activation of PI3K. Paradoxically, mTORC1 negatively regulates the activity of mTORC2 *via* direct inhibition or through S6-kinase. Acute inhibition of mTORC1 by rapamycin and rapalogs can cause feedback activation of mTORC2, which then phosphorylates AKT on serine-473, leading to restoration of PI3K/AKT signaling.

Neurofibromas express several RTK ligands, including neuregulin/heregulin, insulin-like growth factor-1 (IGF-1), and hepatocyte growth factor (HGF) (31). NF1-related PNFs can progress to atypical neurofibromatous neoplasms of uncertain biological potential (ANNUBP), which are hypothesized to be precursor lesions of MPNST. In addition to *NF1* loss, ANNUBP often harbor deletions of *CDKN2A/B* (Cyclin-Dependent Kinase Inhibitor 2A/B). Additionally, MPNSTs frequently acquire mutations in *SUZ12* or *EED*, subunits of the polycomb repressive complex 2 (PRC2) for chromatin remodeling. Loss of PRC2 enhances Ras-driven gene transcription (32). Also, MPNSTs often overexpress several RTKs, including epidermal growth factor receptor (EGFR), HGF receptor (HGFR or MET), platelet-derived growth factor receptor (PDGFR), and insulin-like growth factor-1 receptor (IGF-1R), potentially resulting in autocrine signaling. These aberrantly-activated RTKs can initiate Ras-independent survival signals through mediators, such as the STAT (signal transducers and activators of transcription) transcription factors (33).

The *NF2* gene encodes merlin for moesin, ezrin, and radixin-like protein (17, 18), which shares similarity to the protein 4.1, ezrin, radixin, and moesin (FERM) family of membrane-associated proteins that link cell-surface receptors to the actin cytoskeleton (34). Loss of merlin results in defective cell-cell adhesion *via* destabilizing adherens junctions, which correlates with abnormal activation of focal adhesion kinase (FAK), a downstream target of ECM-binding integrins and MET (35). Merlin negatively regulates ligand-induced internalization and recycling of multiple RTKs, such as EGFR and PDGFR (34) (**Figure 1A**). Cells lacking merlin also show elevated levels of several other RTKs, including IGF-1R, MET, and the EGFR family members ErbB2 and ErbB3 (36, 37). These RTKs activate ERKs and AKT/mTOR, promoting translation and cell growth. Through binding to angiomotin, merlin inhibits p21-activated kinases (PAKs), and PAKs reciprocally phosphorylate and inactivate merlin (38–40). Merlin-deficient cells display deregulated PAK and enhanced invasiveness.

Additionally, merlin suppresses the Hippo/large tumor suppressor kinase 1/2 (LATS1/2)/Yes-associated protein (YAP) pathway (41, 42). Merlin inhibits proteasomal degradation of LATS1/2 by interacting with the E3 ubiquitin ligase CRL4^DCAF1^ in the nucleus (43). Therefore, merlin deficiency is associated with enhanced YAP-dependent gene transcription and cell proliferation. Further, loss of merlin resulted in mTORC1 activation, leading to phosphorylation of ribosomal protein S6 and enhanced cap-dependent protein synthesis *via* inhibition of the eIF4E-binding protein (4E-BP) (44, 45). *NF2*-deficient cells also display mTORC2 activation. Inhibition of mTORC1 by rapamycin amplifies AKT activation by mTORC2 (46, 47).

The molecular mechanisms by which inactivation of the *LZTR1* or *SMARCB1/INI1* gene in addition to the *NF2* gene causes schwannomatosis are not understood. Mutations in *LZTR1* also occur in several cancer types, including ~22% glioblastomas (48). Introducing LZTR1 to *LZTR1*-mutated glioblastoma cells decreases cyclin A and polo-like kinase 1 expression, indicating the importance of LZTR1 in cell cycle progression. As a member of the BTB-kelch superfamily, LZTR1 acts as an adaptor for cullin 3 (Cul3)-containing E3 ubiquitin ligases (27) and facilitates polyubiquitination and degradation of Ras (49). Cul3-containing ubiquitin ligases promote cell differentiation, and *LZTR1*-depleted Schwann cells have gene expression signatures consistent with a demyelinated proliferative phenotype. Additionally, recent evidence indicates that LZTR1-mutant schwannomatosis schwannomas exhibit deregulated VEGF receptor, ErbB3, and ERK signaling (25). SMARCB1 is a component of the SWI/SNF chromatin remodeling complex, which affects the accessibility of genes to transcription factors and RNA polymerases (50). Loss of *SMARCB1* function is associated with deregulated Hedgehog/GLI and WNT/β-catenin signaling. Additionally, SWI/SNF antagonizes PRC function, and SMARCB1 deficiency results in PRC placing repressive histone methylation marks on other tumor suppressor genes, such as *CDKN2A (* 51).

It should be emphasized that although NF1-, NF2-, and schwannomatosis-related tumors have distinct genetic origins, they share downstream activation of several molecular targets (1, 2, 6, 16, 25, 52). Collectively, these deregulated signaling pathways due to tumor suppressor loss in NF-associated tumors provide several targets for therapeutic development.

## Unique Challenges for Developing Medical Therapies for NF

Neurofibromatoses are systemic, life-long diseases with diverse manifestations. Drug safety and high efficacy must be carefully considered for these patients. Due to their anatomical locations, routine biopsies of NF-associated tumors may not be possible. Determination of appropriate endpoints and identification of noninvasive biomarkers to monitor drug pharmacodynamics and predict patient response are of great importance. Owing to their benign nature, NF-associated tumors exhibit variable growth patterns. NF1-related PNFs tend to rapidly expand during childhood but develop a more indolent growth pattern in adults. NF2-associated *VS* grow slowly at one-to-two millimeters per year with occasional increases in growth rates. Therefore, reduction in tumor volume may not be an ideal endpoint, and other metrics, such as time to progression, hearing loss, and pain, should be considered for drug efficacy (53–55). NF2 and schwannomatosis are rare diseases, and patients may be difficult to recruit to clinical trials unless the drugs are effective and well tolerated. For most NF-associated tumors, medication may need to be taken for years. The dosing regimen should be as simple as possible to aid in patient compliance, with a daily oral tablet being ideal. In addition, patients being recruited to clinical trials should be asked whether they are taking any drugs or supplements as there could be toxicities when combining over-the-counter (OTC) with investigational agents.

From identification of targets deregulated in NF-related tumors using traditional and systems biology approaches (56–59), it is anticipated that additional targeted compounds will be successful. The evaluation of these targeted drugs has been the focus of several recent reviews. Below, we summarize the current research on natural compounds in NF-associated tumors (**Table 1**).

**Table 1:** The natural compounds evaluated in NF-related models and their mechanisms of action, preclinical data, and human evaluation.

Natural compound			Preclinical data		Human data			References
Name	Mode(s) of action	Availability	NF cell culture models	NF animal models	Pharmacokinetics	Efficacy studies	Toxicities	
Curcumin	• Inhibits ERK1/2, AKT, NF-κB, and STAT3 • Induces caspase cleavage and MYPT1-pp1δ (merlin phosphatase)	• Turmeric-containing food/drink products • OTC supplements	• HPV-transformed *VS* cells HEI-193 IC_50_ ~10 µM • Primary *VS* IC_50_ 20 µM • *NF2* ^-/-^ Ben-Men-1 IC_50_ 7 µM • *NF1* ^-/-^, *NF1* ^+/-^, and *NF1* ^+/+^ MPNST IC_50_ 25-100 µM	ND	• T_1/2_ < 5min • Poor absorption even after ingesting ~12 g/day	• Supplementation of a Mediterranean, but not Western, diet with curcumin was associated with 30-51% reduction in the number of cNFs	• 12 g/day well-tolerated • Relative lack of adverse effects may be due to poor absorption and short half-life	(60–67)
Calebin-A	• Inhibits ERK1/2, AKT, survivin, histone acetyltransferase	• Turmeric-containing food/drink products	• *NF1^-/^* ^-^, *NF1* ^+/-^, and *NF1^+/^* ^+^ MPNST IC_50_ 12.5-25 µM	• Reduces the growth of *NF1^-/-^* S462TY MPNST xenografts at 100 mg/kg	ND	ND	ND	(64)
Propolis constituents (CAPE and artepillin C)	• Inhibits PAK, NF-κB, and S-phase entry	• OTC supplements, including Bio30 extract	• HPV-transformed *VS* cells HEI-193 IC_50_ ~1.5 µg/mL (Bio30), ~36 µM (CAPE) • *NF1* ^-/-^ S462 MPNST IC_50_ ~8 µg/mL (Bio30) • Primary *VS* and *NF2* ^-/-^ Ben-Men-1 IC_50_ >20 µM (CAPE)	• Bio30 reduces the growth of HEI-193 xenografts and the growth and metastasis of *NF1* ^-/-^ S462 MPNST xenografts at 100 mg/kg	• CAPE: T_max_ ~ 2.7h and T_1/2_ ~ 18.5h	• CAPE: Phase 1 safety clinical trial in healthy patients (NCT02050334)	• Allergic skin rash to CAPE	(63, 68, 69)
Honokiol	• Inhibits ERK1/2 and AKT	• OTC supplements	• HPV-transformed *VS* cells HEI-193 IC_50_ ~26 µM	ND	ND	ND	• Inhibits UDP-glucuronyltransferases • May inhibit the clearance of other drugs	(70, 71)
*trans*-Resveratrol	• Inhibits insulin/IGF1 signaling • Induces autophagy	• Fruits/vegetables • OTC supplements	• Primary *VS* IC_50_ ~20 µM • *NF2* ^-/-^ Ben-Men-1 IC50 >30 µM • *NF2*-positive HBL-52 IC_50_ >50 µM	ND	• C_max_ ~0.6 µM (5 mg dose); ~137 µM (1 g dose) • Extensively metabolized and rapidly degraded	ND	• Well-tolerated orally • High oral doses (>1g daily), associated with GI symptoms • Potential drug interactions, including tamoxifen, docetaxel, and imatinib	(63, 72–75)
Quercetin	• Stabilization of merlin	• Fruits/vegetables • OTC supplements	• HPV-transformed *VS* cells HEI-193 IC_50_ >25 µM • *NF2* ^-/-^ CH157-NM malignant meningioma transfected with mutant merlin protein, ~25 µM for merlin stabilization	ND	• C_max_ ~0.43-3.03 µM	ND	• Well-tolerated when consumed in foods • Mutagenic in the Ames test	(76–81
Sichuan pepper extracts	• Inhibits PAK1 and cyclin D1	• Spice and flavoring agents in various foods • OTC supplements	• *NF1^-/-^* S462 and S-805 MPNST IC_50_ ≥10 µg/mL	• Reduces the growth of *NF1*-deficient MDA-MB-231 breast cancer xenografts at 110 mg/kg	ND	ND	ND	(82)
Cucurbitacins	• Inhibits AKT, ERK1/2, and cyclins • Promotes p21^Waf1^ and tubulin disruption	• Low levels in edible plants and fungi • Higher amounts in bitter squash and melons	• Primary *VS* IC_50_ 250 nM (Cucurbitacin D) • *Nf2^-/-^* mouse schwannoma IC_50_ 750 nM • *NF2* ^-/-^ Ben-Men-1 IC_50_ 200 nM • Primary meningioma IC_50_ 200 nM • *NF1* ^-/-^ ST8814 MPNST ~50 nM (cucurbitacin I)	• Cucurbitacin I reduces the growth of *NF1^-/-^* ST8814 MPNST xenografts at 1 mg/kg	ND	ND	• Severe diarrhea and vomiting, possibly requiring intensive care.	(83–86)
Celastrol	• Inhibits proteasome and NF-κB • Promotes stabilization of merlin	• OTC *T. wilfordii* supplements	• *NF2* ^-/-^ CH157-NM malignant meningioma transfected with mutant merlin protein, ~800 nM for merlin stabilization	ND	ND	ND	• Hepatic, nephrotic, and cardiovascular toxicity • Inhibits CYP-450 enzymes with potential drug interactions • Impedes mitochondrial respiration	(77, 87, 88)
Goyazensolide	• Inhibits AKT, cyclins, and NF-κB	• Not commercially available for consumption	• Primary *VS* IC_50_ <4 µM • *Nf2^-/-^* mouse schwannoma cells, IC_50_ 0.9 µM • *NF2* ^-/-^ Ben-Men-1 IC_50_ 1 µM • Primary meningioma IC_50_ <4 µM	• Therapeutic doses toxic in mice	ND	ND	ND	(86, 89)
DAW22	• Inhibits AKT, ERK1/2, and β-catenin	• Not commercially available for consumption	• *NF1* ^-/-^, *NF1* ^+/-^, and *NF1* ^+/+^ MPNST IC_50_ ~30-47 µM	• Modestly reduces the growth of *NF1* ^+/+^ STS26T MPNST xenografts at 60 mg/kg	ND	ND	ND	(90)
Sulforaphane	• Inhibits NF-κB	• Plants and vegetables, including broccoli, cabbage, Brussels sprouts • OTC supplements	• HPV-transformed *VS* cells HEI-193 IC_50_ > 10 µM • Primary *VS* IC_50_ > 20 µM • *NF2* ^-/-^ Ben-Men-1 IC_50_ 8 µM	• Modest reduction in *Nf2* ^-/-^ SC4 mouse Schwann cell tumor weight at 25 mg/kg	• C_max_ ~200 nM	ND	• Well-tolerated orally	(63, 91–94)
Cannabidiol	• Binds to CB1 and CB2 • Modulates other ion channels and neuroreceptors	• Oral suspension (Epidiolex) FDA-approved for epilepsy • OTC supplements, e-cigarettes, and food/drink products	ND	ND	• High volume of distribution (32 L/kg) • C_max_: ~2.2 µM (IV); ~0.008-0.011 µM (PO); ~0.35 µM (smoking); ~0.03 µM (nebulizer) • Bioavailability: ~6% (PO); ~31% (smoking)	• Case study of NF1 patient with PNF: sublingual CBD oil appeared to help with chronic pain and mood control	• Drowsiness, dizziness • Oral route associated with more psychoactive effects	(95–99)
Ivermectin	• Inhibits PAK1 and Raf1	• Tablets/capsules prescribed for parasitic infections	• HPV-transformed *VS* cells HEI-193 IC_50_ ~ 5 µM	• CNS toxicity in mice with MDR1 knockout (topical spray) and in dog breeds with compromised MDR1 function (anti-parasitic medications)	• C_max_ <100 nM	ND	• Potential drug-drug and drug-food interactions through effects on CYP3A4 and MDR1	(100–102)
Silvestrol	• eIF4A inhibitor • Inhibits prohibitins	• Not commercially available for consumption • Source plants are endangered	• Primary *VS* IC_50_ 15 nM • *Nf2^-/-^* mouse schwannoma IC_50_ 70 nM • *NF2* ^-/-^ Ben-Men-1 IC_50_ 10 nM • Primary meningioma 25 nM • *NF1* ^-/-^ and *NF1* ^+/+^ MPNST IC50 ≤ 70 nM	• Suppresses the growth of mouse *Nf2^-/-^* schwannoma allografts and human meningioma and *NF1* ^-/-^ MPNST xenografts at 1.5 mg/kg • Bioavailability (mice): IP ~100%, oral ~1.7% • Lung toxicity in dogs (IV)	ND	ND	ND	(63, 103–110)
Roc and DDR	• eIF4A inhibitor • Inhibits Prohibitins	• Not commercially available for consumption • Source plants are endangered	• Primary *VS* IC_50_ 25 nM (Roc); 8 nM (DDR) (unpublished) • *Nf2^-/-^* mouse schwannoma IC_50_ 10 nM (DDR) • *NF2* ^-/-^ Ben-Men-1 IC_50_ 15 nM (Roc); 5 nM (DDR) • *NF1* ^-/-^, *NF1* ^+/-^, and *NF1* ^-/-^ MPNST IC_50_ 12-50 nM (Roc); 5-15 nM (DDR)	• Suppresses the growth of human *NF1* ^-/-^ ST8814 and patient-derived MPNST xenografts (IP at 3 mg/kg and PO at 1.2 mg/kg) • C_max_ of Roc in mice ~11 µM (IV); ~4 µM (IP); ~ 0.8 µM (PO) • Bioavailability (Roc in mice): ~100% (IP), ~ 1.7% (PO) • Well-tolerated in dogs (IV)	ND	ND	ND	(104, 106, 108, 110–113)
Annonacin	• Mitochondrial complex I inhibitor	• Fruits and leaves from soursop, custard apples, paw paws, and related plants in Annonaceae • OTC supplements	ND	ND	ND	ND	• Neurotoxicitiy to dopaminergic neurons • Atypical Parkinson's disease associated with high consumption of soursop	(114–118)

ND, Not determined; CBD, cannabidiol; CAPE, caffeic acid phenethyl ester; DDR, didesmethylrocaglamide; Roc, rocaglamide; HPV, human papillomavirus; OTC, over-the-counter; IP, intraperitoneal injection; IV, intravenous injection; PO, per oral; Cmax, maximum concentration; T1/2, half life; Tmax, time to Cmax; CNS, central nervous system; GI, gastrointestinal.

## Natural Compounds for Preventative Purposes and Potential Treatments of NF

Natural compounds from terrestrial microbes, higher plants, and marine organisms have been studied as cancer chemotherapeutic agents for several decades. Over the last 40 years, ~50% of FDA-approved drugs are natural products, natural product derivatives, or synthesized compounds based on pharmacophores originally identified in natural products (119). For example, the DNA-intercalating anti-neoplastic agents anthracyclines are made by *Streptomyces* bacteria. The microtubule-disruptor Taxol^®^ (paclitaxel) was originally isolated from the bark of Pacific yew. Due to the slow-growing nature of this plant, Taxol is manufactured semi-synthetically from a precursor or produced by plant cell culture. The topoisomerase I inhibitor camptothecin was obtained from *Camptotheca acuminata* (happy tree). Several camptothecin analogs, including topotecan (Hycamtin^®^) and irinotecan (Camptosar^®^), have been synthesized. Trabectedin (Yondelis^®^, ecteinascidin 743, ET-743), which interferes with transcription and related processes, was discovered from extracts of the sea squirt *Ecteinascidia turbinata* and later found to come from *Candidatus Endoecteinascidia frumentensis*, a γ-proteobacterium living in symbiosis with the sea squirt. Since natural compounds tend to have diverse structural complexity and may inhibit molecules previously thought to be untargetable, the U.S. National Cancer Institute has divisions focused on drug discovery within the natural product space aiming to identify agents that inhibit difficult targets. Also, natural compounds have served valuable roles as probes to delineate the signaling pathways important for cell growth (103).

**Figure 2:**
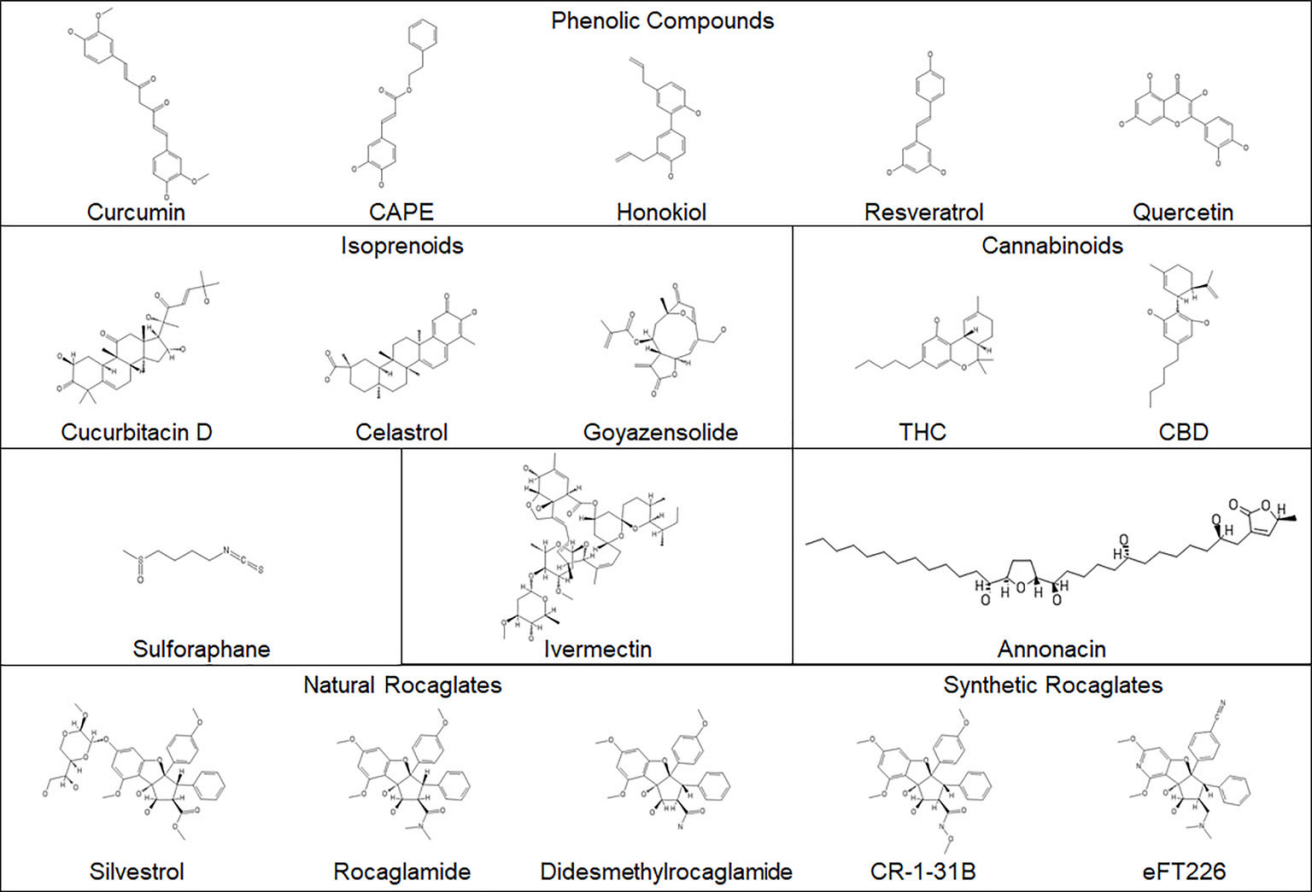
The structures of the natural compounds evaluated in NF-associated disease. Synthetic rocaglates are included for comparison.

In addition to anti-neoplastic activity, natural compounds may possess antioxidant and anti-inflammatory properties and are particularly attractive as adjunct therapies for patients with tumor predisposition syndromes, including NF. Due to limited treatment options, NF patients often take them as dietary supplements for health enhancement purposes. Although much research has been conducted to evaluate anti-tumor activities of natural products, most studies only report their *in vitro* effects. Sometimes high doses are used, which may not be achievable or even desirable *in vivo*. At high concentrations, small molecules may cause off-target effects, unwanted redox activities, and anomalous plasma membrane permeability. Some natural dietary supplements are labeled as safe with health benefits, but these claims have not been validated. Many supplements are crude extracts, containing a mixture of compounds with the active components unknown, and lot-to-lot variability could contribute to irreproducible results. Compound sourcing may affect the activity of natural products. Also, some supplements may have dangerous contaminants, such as heavy metals, or may not contain the claimed ingredients. Therefore, dietary supplements should be carefully verified for overall chemical composition and safety (120). For purified single-chemical entity natural products that may be developed into new therapies for NF, it is important that their biological activities be carefully investigated and that they be treated as tractable hits, defined as compounds with rational structure-activity relationships. Here we summarize the natural compounds (**Figure 2**) that have been evaluated in NF-related tumor cell and animal models.

### Phenolic Compounds

Characterized by the presence of one or more phenol functional groups, these compounds comprise flavonoids, usually found in herbs, citrus fruits, and other plants, and non-flavonoids, also present in plants and including the curcuminoids in turmeric (121). However, phenolic compounds may be difficult to develop into drugs as many nonspecifically perturb cell membrane and alter protein function. The sections below summarize several phenolic compounds that have been evaluated in NF-related tumor models.

**(i) *Curcumin and other curcuminoids*.** The diarylheptanoid curcumin is isolated from turmeric (*Curcuma longa)* and comprises 2-5% of the plant rhizome. Turmeric is commonly used in Indian cooking and is a component in Ayurvedic medicine for treating infections, inflammation, and other chronic conditions. Curcumin possesses anti-inflammatory properties through inhibition of the NF-κB pathway and suppression of phospho-STAT3 in cancer cells at relatively high IC_50_ (50% inhibitory concentration; ~20µM) (60, 61). However, curcumin has also been shown to induce apoptosis by increasing pro-inflammatory oxidative damage. Using HEI-193, a human papillomavirus oncogene-transformed NF2-associated *VS* cell line, curcumin inhibits colony formation at ~10µM by downregulating the ERK, AKT, and NF-κB pathways while increasing free radical-mediated apoptosis (62) (**Table 1**). These results suggest cell context-dependent effects of curcumin. Also, curcumin enhances the expression of heat shock protein 70 (HSP70), a molecular chaperone associated with drug resistance. Combining curcumin with the pan-HSP inhibitor KNK437 yields synergistic growth inhibition in HEI-193 cells. The use of HEI-193 as an NF2-related cell model is of concern as viral oncogene transformation alters growth behavior of benign schwannoma cells. Other NF2-associated schwannoma models should be evaluated to confirm the findings.

Previously, we found that the IC_50_ value of curcumin was ~20µM in primary *VS* cells (63) and ~7µM in *NF2*-deficient Ben-Men-1 meningioma cells. Curcumin also inhibits proliferation of *NF1*-deficient MPNST cells at IC_50_ values of 25-100µM (64). A related curcuminoid, calebin-A, reduces cell growth at IC_50_ values ranging from 12.5-25µM and decreases the levels of phospho-AKT and survivin in the same MPNST cell lines. Calebin-A shows modest anti-tumor activity in an *NF1*-deficient MPNST xenograft model when dosed at 100mg/kg (**Table 1**). A dietary study in a small number of NF1 patients fed a Mediterranean diet supplemented with curcumin reported a 30-51% reduction in the number of CNFs, while a Western diet with the same supplement did not show any tumor inhibition (65). A larger study with well-defined objective outcomes is required to draw firm conclusions about the efficacy of curcumin in NF1 patients.

Curcumin has a short half-life in aqueous solution (<5 minutes), making it imperative that experiments be rigorously controlled to ensure the observed treatment effects are due to curcumin but not its degradation products (66). The instability of curcumin along with poor bioavailability may explain why many early trials did not show any clinical benefits. To increase bioavailability, alternative delivery approaches have been sought, including protein nanoparticles, liposomal formulations, and erythrocytes coated with porous nanoparticles (66, 67). It remains to be seen if these strategies could improve the efficacy of curcumin.

**(ii) *Propolis constituents*.** Also called bee glue, propolis is found in beehives and has been used since the ancient Egyptian civilization for its anti-inflammatory, anti-bacterial, and wound-healing properties. It comprises an admixture of different chemicals with wide variability in composition depending upon factors, such as the regional flora, climate, and preparation method (68). Therefore, studies using crude propolis extracts are highly liable to lot-to-lot variabilities in their biological effects. Chinese red propolis inhibits VEGF expression and contains at least 12 components, including the phenolic ester, caffeic acid phenethyl ester (CAPE), and the flavonoid, kaempferol. Turkish propolis induces G_1_ arrest and apoptosis in cancer cells and contains six major constituents, including caffeic acid and CAPE. Polish propolis inhibits S-phase entry, decreases cell viability, and contains multiple flavonoids. Bio30 is a water-miscible extract of New Zealand propolis, which contains multiple phenolic compounds including CAPE, and suppresses the growth of HEI-193 schwannoma and *NF1*-deficient S462 MPNST cells (69) (**Table 1**). Among these propolis specimens, a common constituent is CAPE. The IC_50_ of this compound in HEI-193 schwannoma cells was ~36 µM. We also found that CAPE has little growth-inhibitory activity in *VS* and meningioma cells at doses <=20µM (63). It is not known whether it is possible to achieve this high concentration of CAPE in humans.

**(iii) *Honokiol*.** As a biphenolic lignan from the bark of *Magnolia* trees, honokiol is commonly consumed as an ingredient in herbal tea preparations for treating anxiety in Asia and possesses anti-tumor activity (70). In HEI-193 schwannoma cells, honokiol inhibits proliferation by decreasing phospho-AKT and ERK1/2 at an IC_50_ of ~26µM (71) (**Table 1**). However, this compound has not been tested in other NF2- or NF1-related models. Also, honokiol crosses the blood-brain barrier, but its oral bioavailability and plasma half-life are low (70). Several approaches, including encapsulating honokiol in nanoparticles or liposomes, are being used to enhance these properties.

**(iv) *trans-Resveratrol*.** This stilbenoid was isolated originally from white hellebore, but is found widely in various fruits and nuts, including grapes, apples, and pistachios (72). It has beneficial metabolic effects in mouse models of diet-induced diabetes by negatively modulating insulin and IGF-1 signaling. It inhibits cell proliferation by reducing the levels of cyclin D, Hippo-YAP, and β-catenin. In addition, resveratrol at doses of 5mg or less per day reduces the number of adenomas by ~40% in a mouse model of high-fat diet-induced colorectal carcinoma by promoting autophagy, while a higher dose of ~1g per day shows less inhibitory effects. These results suggest that resveratrol may have chemo-preventive effects (73). Pharmacokinetic studies indicate that a single dose of resveratrol of 5mg or 1g reaches peak plasma concentrations of 0.6 and 137µM, respectively. It is generally accepted that resveratrol at doses ≤1g can be taken long-term, but higher doses (e.g., 2.5 and 5 g) can cause gastrointestinal symptoms (74). In human *VS* cells and *NF2*-deficient Ben-Men-1 cells, resveratrol exhibits moderate antiproliferative activity at doses up to 20µM (63). In *NF2*-expressing HBL-52 meningioma cells, the effective dose of resveratrol to induce apoptosis is >50µM (75). Therefore, additional studies in NF-related animal models are needed to determine if resveratrol exhibits anti-tumor effects at tolerable doses.

**(v) *Quercetin*.** The pigmented pentahydroxylated flavone quercetin is widely found in oaks (genus *Quercus*), herbs, fruits, and vegetables. It induces apoptosis at a relatively high dose and moderately reduces cancer cell growth (76). Quercetin inhibits the growth of HEI-193 schwannoma cells and stabilizes the expression of mutant merlin proteins in *NF2*-deficient CH157-NM malignant meningioma cells at doses ≥25µM (77); however, these doses are not possible to be reached in humans (**Table 1**). Additionally, it is known to aggregate and promiscuously bind to proteins, making it less therapeutically effective and potentially toxic (78–81). The known toxic effects of quercetin include mutagenicity, prooxidant activity, and mitochondrial toxicity.

**(vi) *Sichuan pepper extracts*.** The Sichuan peppercorns harvested from the seeds of the aromatic spiny shrub *Zanthoxylum piperitum* are used in East Asian cuisine and traditional Chinese medicine. Peppercorn extracts inhibit proliferation of *NF1*-deficient MPNST and MDA-MB-231 breast cancer cells by reducing PAK1 activation and cyclin D_1_ levels (82) (**Table 1**). These extracts also suppress MDA-MB-231 xenografts, but resistant cell populations rapidly developed during treatment. Moreover, the constituents in Sichuan pepper extracts responsible for the antiproliferative effects have not been determined.

### Isoprenoids

Terpenoids and steroids are isoprene-derived, with terpenes being a class of naturally-occurring hydrocarbons originally named for their discovery in turpentine, a resin distilled from conifer sap. Isoprenoids are the largest group of natural products. Although they are mainly found in plants, some classes, such as steroids, are common in animals. In plants, terpenoids are often found as aromatic compounds that play important roles in signal transduction and act as a defense against herbivores (122). With great structural diversity, terpenoids are made up of repeating units of the C_5_-hydrocarbon isoprene and classified based on the number and structural organization of isoprene units. Although the words terpenes and terpenoids are often used interchangeably, terpenoids properly refer to modified terpenes with additional oxygenated functional groups.

**(i) *Cucurbitacin*.** Originally isolated from the squash family (Cucurbitaceae), cucurbitacins are tetracyclic triterpenoids with a steroidal skeleton (83). These compounds are also found in several other plants and mushrooms. They often have glycosidic linkages and are classified into multiple variants according to their side chains. Cucurbitacins are contained in traditional Asian remedies for treating viral diseases and inflammatory conditions. They exhibit antiproliferative effects by disrupting microtubule polymerization. Also, they inhibit cancer cell growth by decreasing phospho-AKT and phospho-ERK, increasing the levels of p21^WAF1^, and promoting apoptosis (84). Cucurbitacin I effectively inhibits proliferation of *NF1*-null MPNST cells and induces apoptosis by decreasing STAT3 signaling (85) (**Table 1**). We showed that cucurbitacin D has growth-inhibitory activity against *Nf2*-null mouse schwannoma and human Ben-Men-1 cells at sub-micromolar IC_50_ concentrations by decreasing the expression of cyclins and phospho-AKT, leading to G_2_/M arrest (86). Despite their anti-tumor activity, cucurbitacins may cause gastrointestinal toxicity and have a low therapeutic index, which hamper their further clinical development (84).

**(ii) *Celastrol*.** Originally isolated from *Tripterygium wilfordii* (thunder god vine), celastrol (tripterine) is a pentacyclic triterpenoid, which exhibits anti-obesity, antioxidant, anti-inflammatory, and anti-tumor effects (87). It inhibits NF-κB signaling and multiple other pathways and reduces proliferation and invasion of cancer cells with an IC_50_ of ~2µM. Celastrol impedes degradation of the merlin protein in malignant meningioma cells carrying a missense mutation in the *NF2* gene at ~1µM (77) (**Table 1**). However, celastrol has a problematic *ortho*-quinone methide functional group that possesses redox activity and is reactive promiscuously with the sulfur nucleophiles present in the active sites of several enzymes, including metabolic coenzymes, needed by normal cells. These features may explain the serious adverse effects on hepatic, renal, reproductive, and cardiovascular systems reported after consuming *T. wilfordii* supplements (88).

**(iii) *Goyazensolide*.** Isolated from *Piptocoma rufescens* (velvetshrub) and other members of the sunflower family (Asteraceae), goyazensolide is a sesquiterpene lactone. It was identified initially as an anti-schistosomal agent and has antiproliferative activity by inhibiting NF-κB expression and inducing apoptosis (89). We found that goyazensolide suppresses proliferation of *Nf2* ^-/-^ mouse schwannoma cells and *NF2*-deficient human meningioma cells at IC_50_ doses of ~1µM and less effectively in primary human *VS* and meningioma cells (86) (**Table 1**). These growth-suppressive effects appear to be due to decreased expression of AKT and cyclins, followed by G_2_/M arrest. Unfortunately, goyazensolide was too toxic in mice at therapeutic doses for further development.

**(iv) *DAW22*.** This sesquiterpene coumarin was isolated from the roots of *Ferula ferulaeoides*, a member of the carrot family (Apiaceae). It is antiproliferative and pro-apoptotic in sporadic and NF1-associated MPNST cells but at relatively high IC_50_ doses (30-47µM) (90) (**Table 1**). Also, it only modestly reduces tumor growth in *NF1*-expressing MPNST-bearing mice at the dose of 60mg/kg. The effects of DAW22 in NF2-related tumor cell and animal models have not been investigated.

### Sulforaphane

Frequently found as a glycosidic precursor in cruciferous vegetables of the mustard family (Brassicaceae), sulforaphane is a sulfur-containing member of the isothiocyanate compound class (91). It has anti-inflammatory and anti-neoplastic activities in several types of cancer cells, partly *via* inhibiting NF-κB. In HEI-193 schwannoma cells, sulforaphane has growth-inhibitory effects at IC_50_ >10µM (92) (**Table 1**). We also found that it inhibited the growth of primary *VS* and meningioma cells at IC_50_ >20µM (63). Due to its short half life (93), the plasma concentrations of sulforaphane in humans peak at ~200nM (94). These results suggest that it may not be very potent against NF2-related tumors. However, sulforaphane is generally safe (93), improved formulation and delivery methods will be required to reach a therapeutic level.

### Cannabinoids

*Cannabis sativa*, called marijuana or hemp, is used in traditional Chinese and Ayurvedic medicine, but in the West, more well-known for recreational purposes. Although *Cannabis sativa* varieties synthesize >100 different cannabinoids, two compounds, Δ^9^-tetrahydrocannabinol (THC) and cannabidiol (CBD), are primarily studied for their clinical effects. *Cannabis* extracts and individual cannabinoids are increasingly used in patients suffering from glaucoma, neuropathic pain, and cancer (95, 96). THC is known for its appetite stimulation and psychoactive properties and is a Schedule 1-controlled substance. Dronabinol (Marinol^®^, Syndros^™^), a synthetic THC analog, is FDA-approved for treating HIV-induced appetite loss and chemotherapy-related nausea and vomiting. Nabiximols (Sativex^®^), a 1:1 THC : CBD extract, is approved in ~30 countries. for multiple sclerosis-related symptoms. Cannabinoids may have anti-neoplastic activity through their effects on endocannabinoid receptors (97). The synthetic THC analog, WIN-55212-2, is an endocannabinoid receptor agonist and induces G_1_ cell cycle arrest (123). In some contexts, cannabinoids may be pro-tumorigenic as 100-300nM THC enhances DNA synthesis in cancer cells by endocannabinoid receptor-mediated EGFR transactivation (124). However, it should be noted that overdoses of THC may cause acute intoxication, tachycardia, aboulia, and psychosis. Also, THC and related analogs have not been assessed in NF-related cell and animal models.

In contrast, CBD is non-psychoactive and has analgesic, anxiolytic, and anticonvulsant properties (95, 96). In the U.S., chemically-synthesized CBD is legally sold as an over-the-counter supplement. Clinical trials suggest that CBD is overall well-tolerated with drowsiness and dizziness being the main adverse effects (96) (**Table 1**). The FDA approved Epidiolex^®^, a plant-derived CBD, to treat Lennox-Gastaut and Dravet syndromes, two rare forms of severe epilepsy. In addition, CBD may have helped control neuropathic pain and mood disorders in an NF1 patient with PNFs (98). A larger study is needed to confirm these findings. CBD preparations are sold in a wide variety of formulations, and the purity and safety of different products are unclear. Additional rigorous clinical examinations should be conducted to validate analgesic qualities, pharmacokinetics, and long-term safety (99).

### Ivermectin

The anti-parasitic avermectins were originally isolated from *Streptomyces avermitilis*. Subsequently, a semi-synthetic derivative of avermectin, ivermectin, was developed for veterinary use to treat parasite infestations and was later approved by the FDA to treat river blindness and other nematode infections in humans (100, 101). Ivermectin is well tolerated and only causes mild toxicity even when taking ten times the FDA-approved dose. It has anti-tumor activity in various types of cancer. It inhibits proliferation of HEI-193 schwannoma cells at an IC_50_ of ~5µM by blocking PAK1 and decreasing phospho-Raf (102) (**Table 1**). However, the FDA-approved therapeutic dose of ivermectin in humans only reaches plasma concentrations of <100nM (100). Also, ivermectin is a substrate for the multidrug resistance 1 (MDR1) transporter, which prevents the drug from reaching high concentrations in the brain (100, 101). Thus, this drug is not likely to be effective against NF2-related *VS* and meningiomas.

### Silvestrol, Rocaglamide, and Didesmethylrocaglamide

Rocaglates, also called flavaglines, are a large family of cyclopenta[*b*]benzofurans synthesized by tropical trees of the *Aglaia* genus in the mahogany family (Meliaceae) (104). Among this group of natural compounds, rocaglamide (also known as rocaglamide A or RocA; **Figure 2**) was first found to possess antileukemic activity (111) but was not further characterized biologically for some years due to its scarcity. Subsequently, a few other rocaglates with anti-proliferative activity were identified, including silvestrol which was the first flavagline with an unusual sugar-like dioxanyl ring discovered in *Aglaia foveolata (* 105). Silvestrol inhibits proliferation of a variety of cancer cell lines at low nanomolar concentrations similar to camptothecin and paclitaxel (104). Acting as inhibitors of the eukaryotic translation initiation factor 4A (eIF4A), an RNA helicase (106), silvestrol and rocaglamide bind eIF4A and lock it onto purine-rich sequences in the 5’-untranslated region (UTR) of certain mRNAs, leading to translation inhibition (107, 112) (**Figure 1B**). Rocaglates may also bind to prohibitins, resulting in inhibition Raf/ERK signaling (108).

We have shown that MPNST, *VS*, and meningioma tumors frequently overexpress the eIF4F components, including eIF4A (63, 109). Genetic depletion of eIF4A *via* RNA interference and pharmacological inhibition by silvestrol effectively suppress proliferation of *NF2*-deficient tumor and *NF1*-deficient MPNSTs cells (**Table 1**). As an eIF4A inhibitor, silvestrol reduces the protein levels of multiple cyclins and oncogenic kinases, including AKT, ERK, and FAK, leading to G_2_/M arrest and apoptosis. Also, it profoundly suppresses tumor growth of *Nf2^-/-^* schwannomas and *NF1^-/-^* MPNSTs. However, a toxicology study in dogs revealed that silvestrol caused lung damage (https://dtp.cancer.gov/publications/silvestrol_rocaglamide_studies.pdf). Consequently, its further clinical development was suspended (103).

By side-by-side comparing 10 silvestrol-related rocaglates lacking the dioxanyl ring (110), we identified rocaglamide and didesmethylrocaglamide (also called RocB) with growth-inhibitory activity comparable to silvestrol in MPNST, schwannoma, and meningioma cells at low nanomolars (113) (**Table 1**). Both rocaglamide and didesmethylrocaglamide reduce expression of multiple oncogenic kinases IGF-1R, AKT, and ERKs while simultaneously inducing DNA damage response, caspase cleavage, and apoptosis. Interestingly, rocaglamide exhibited 50% oral bioavailability and was not susceptible to multi-drug resistance-1 efflux. When delivered by oral gavage or intraperitoneal injection, rocaglamide potently suppressed tumor growth in an orthotopic MPNST model. Most importantly, rocaglamide was well tolerated in mice and did not induce pulmonary toxicity in dogs. Furthermore, both rocaglamides exhibited strong anti-tumor effects against other sarcomas, including osteosarcoma, Ewing sarcoma, and rhabdomyosarcoma. These results warrant a clinical trial to evaluate these rocaglamides in patients with sarcomas and those afflicted by NF. It should also be mentioned that the synthetic rocaglates have also been developed that retain the core scaffold responsible for eIF4A inhibition, while incorporating side chain modifications to optimize pharmacokinetic and pharmacodynamic properties. One such compound, (-)-CR-1-31B prolongs survival of mice bearing pancreatic adenocarcinoma allografts (125). Another synthetic rocaglate-like compound, eFT226 (zotatifin), has anti-tumor activity against several fibroblast growth factor receptor- and ErbB2-driven cancers (126) and has recently entered a phase 1/2 clinical trial in patients with K-Ras- or RTK-driven advanced solid tumors (ClinicalTrials.gov Identifier: NCT04092673).

### Annonacin

Also called guyabano or graviola, soursop is the fruit of *Annona muricata*, a member of the custard apple family (Annonaceae). With its pleasant aroma, soursop is used to make juices and as a flavoring agent (**Table 1**). Extracts from fruits and leaves of *Annona muricata* are reported to have anti-tumor activity against multiple tumor types, including anecdotally shrinking NF2-associated *VS* (114–116). One active component with anti-proliferative activity in soursop is annonacin, an acetogenin. However, this compound inhibits mitochondrial complex I, elicits severe neurotoxic effects, and excessive consumption of *Annona* plants and supplements are associated with atypical Parkinson’s disease (117). These serious adverse effects prevent it from further development as an anti-tumor agent (118).

Additionally, several other natural compounds may be of interest to NF patients. Silibinin, the main flavonolignan component in the extract of milk thistle (*Silybum marianum*) seeds, is used to treat hepatotoxicity caused by poisoning from the death cap mushroom *Amanita phalloides*. It inhibits lung cancer cell proliferation by suppressing AKT and ERK activation (127). Gingerol, an alkylphenol found in ginger, decreases the growth of breast cancer cells by lowering the expression of EGFR and β1-integrin (128). Shikonin, a naphthoquinone pigment found in the root of *Lithospermum erythrorhizon* in the borage family (Boraginaceae), is used in traditional Chinese medicine for treating inflammatory diseases. It suppresses leukemia cell growth by decreasing phospho-AKT and ERKs (129). *Angelica sinensis*, commonly known as dong quai (danggui, dang’ui), is an herb in the carrot family (Apiaceae) used in traditional Asian medicine for reproductive disorders. An active component in dong quai is the γ-lactone *N*-butylidenephthalide, which inhibits proliferation of gastric carcinoma cells by increasing the levels of REDD1 (regulated in development and DNA damage responses 1), a negative regulator of the mTOR pathway (130). Genistein is an isoflavone found in soy-based foods, such as soymilk. It induces apoptosis, reduces tumor vascularity, and suppresses metastasis by inhibiting cyclins and AKT activation (131). Epigallocatechin gallate is the most abundant catechin ester in green tea (*Camellia sinensis*). It decreases phosphorylation of PI3K and AKT and reduces IGF-1R levels in some cancer cells (132). Since these natural compounds inhibit the signaling pathways frequently activated in NF-associated tumors, it will be interesting to see whether they have anti-tumor effects in these tumors.

## Conclusion

NF are characterized by multiple nervous system tumors and other non-tumoral manifestations. Surgery and/or radiation are the standard of care to control the tumor burden but often incur significant morbidities. The recent approval of the MEK inhibitor selumetinib to treat NF1-associated PNF suggests that medications targeting specific NF signaling pathways can be successful. However, combining selumetinib with other targeted agent(s) will be needed to achieve a cure. Although not FDA-approved, bevacizumab is used off-label for NF2 patients, with some of them experiencing tumor reduction and improved hearing. Recently, using traditional and systems biology approaches (56–59), several targeted compounds and compound combinations with anti-tumor effects in NF-related models have been identified, and some are being evaluated in humans.

Natural compounds have been investigated as potential cancer therapies for several decades, and many are on the WHO’s List of Essential Medicines. Many patients with NF-related tumors take natural products as supplements in the hope of reducing tumor growth. While several natural compounds can inhibit signal transduction pathways deregulated in NF-associated tumors (**Figures 1** and **2**), most have only been tested in cell culture models and exhibit high IC_50_ values that may not be achievable *in vivo* (**Table 1**). In some cases, the cell culture and animal models used do not accurately reflect the pathogenesis of NF tumors. Therefore, the published data should be interpreted cautiously, with patients consulting their physicians before taking any natural compounds. Of the natural compounds that demonstrated potent anti-tumor activity in both NF-related models, the eIF4A inhibitors rocaglamide and didesmethylrocaglamide effectively block the expression multiple oncogenic kinases with good bioavailability and toxicity profiles and are expected to enter clinical trials in the future.

## Author Contributions

AA: project conception and manuscript writing. JO: project conception and manuscript writing, DW: project conception. AK: project conception, and L-SC: project conception and supervision and manuscript writing and authorship. All authors contributed to the article and approved the submitted version.

## Funding

This study was supported by grants from the Galloway Family, Advocure NF2, CancerFree KIDS, Sunbeam Foundation, NF2 Biosolutions, Department of Defense W81XWH-18-1-0547, and National Institute of Neurological Disorders and Stroke R01NS113854 to L-SC, the Department of Defense W81XWH-14-1-0167 to DW, and National Cancer Institute P01CA125066 to AK and P30CA16058 to The OSU Comprehensive Cancer Center. The funder was not involved in the study design, collection, analysis, interpretation of data, the writing of this article or the decision to submit it for publication.

## Conflict of Interest

L-SC and AK: Patent: U.S. Provisional Application No. 19/55304 Anticancer rocaglamide derivatives. DW: Consultant: CereXis.

The remaining authors declare that the research was conducted in the absence of any commercial or financial relationships that could be construed as a potential conflict of interest.

## Publisher’s Note

All claims expressed in this article are solely those of the authors and do not necessarily represent those of their affiliated organizations, or those of the publisher, the editors and the reviewers. Any product that may be evaluated in this article, or claim that may be made by its manufacturer, is not guaranteed or endorsed by the publisher.
